# Relief Alternatives during Resuscitation: Instructions to Teach Bystanders. A Randomized Control Trial

**DOI:** 10.3390/ijerph17155495

**Published:** 2020-07-30

**Authors:** María José Pujalte-Jesús, César Leal-Costa, María Ruzafa-Martínez, Antonio Jesús Ramos-Morcillo, José Luis Díaz Agea

**Affiliations:** 1 Faculty of Nursing, Universidad Católica de Murcia UCAM, 30107 Murcia, Spain; mjpujalte@ucam.edu; 2 Faculty of Nursing, University of Murcia, 30107 Murcia, Spain; 3 Faculty of Nursing, Catholic University of Murcia, 30107 Murcia, Spain; jluis@ucam.edu

**Keywords:** cardiopulmonary resuscitation, chest compression, method, experiential learning, observation, CPR

## Abstract

To analyze the quality of resuscitation (CPR) performed by individuals without training after receiving a set of instructions (structured and unstructured/intuitive) from an expert in a simulated context, the specific objective was to design a simple and structured CPR learning method on-site. An experimental study was designed, consisting of two random groups with a post-intervention measurement in which the experimental group (EG) received standardized instructions, and the control group (CG) received intuitive or non-standardized instructions, in a public area simulated scenario. Statistically significant differences were found (*p* < 0.0001) between the EG and the CG for variables: time needed to give orders, pauses between chest compressions and ventilations, depth, overall score, chest compression score, and chest recoil. The average depth of the EG was 51.1 mm (SD 7.94) and 42.2 mm (SD 12.04) for the CG. The chest recoil median was 86.32% (IQR 62.36, 98.87) for the EG, and 58.3% (IQR 27.46, 84.33) in the CG. The use of a sequence of simple, short and specific orders, together with observation-based learning makes possible the execution of chest compression maneuvers that are very similar to those performed by rescuers, and allows the teaching of the basic notions of ventilation. The structured order method was shown to be an on-site learning opportunity when faced with the need to maintain high-quality CPR in the presence of an expert resuscitator until the arrival of emergency services.

## 1. Introduction

The out-of-hospital cardiorespiratory arrest (OHCA) is a frequent health problem in developed countries, and only a small percentage of the victims receive cardiopulmonary resuscitation (CPR) by the bystanders [1]. Early and high-quality resuscitation maneuvers can double, or even quadruple, survival [2], however, CPR training of the general population is scarce. The current protocols differ depending if they are directed towards professionals or laypersons, and the health services continue exploring alternatives to improve bystander CPR rates when witnessing out-of-hospital cardiorespiratory arrests. Among these alternatives, we find mass training and telephone-based CPR, to such an extent that in 2015, the European resuscitation guidelines [3] recognized the important role of dispatcher-assisted CPR in the diagnosis and providing of telephone-assisted, early cardiopulmonary resuscitation.

From that point on, the attempts to combine standardized communication in CPR have increased [4,5], with supporters [6] and critics [7] until 2019, when the International Liaison Committee on Resuscitation (ILCOR) [8] recommended that the dispatchers provide instructions to the bystanders. From that point on, many research studies provided information about the increased survival rate [9,10], and affirmed that the provision of dispatch cardiopulmonary resuscitation instructions, instead of no instructions at all, improved the results from cardiopulmonary arrest [11].

However, all of these studies suggest that the clinical results after out-of-hospital cardiac arrest have a greater possibility of improving when dispatcher assistance is available; the scientific societies identify, in their knowledge gap, the preferred CPR instruction sequence for Dispatcher-Assisted Cardiopulmonary Resuscitation (DA-CPR) [8], because an optimal sequence of orders is not yet available for those who are limited to receiving instructions from emergency services personnel. At present, the ILCOR is still seeking the best evidence through the Consensus on Science with Treatment Recommendations (CoSTR) [12].

Between the CPR observed by an expert and telephone-based CPR, we find real-life situations where only one expert/healthcare worker performs resuscitation maneuvers surrounded by people without training, but who could play an important role in maintaining high-quality CPR if they could learn how to do so on-site. These possible scenarios would need to have certain essential elements, described by Bandura [13,14], when learning a skill through observation: attention and motivation. If motivated people were available who are willing to relieve the expert resuscitator, then this expert could provide the necessary instructions to teach CPR to the bystanders for the benefit of the patient. The exhaustion of a single resuscitator could reduce the possibilities of maintaining high-quality CPR until the arrival of the emergency services.

Recent studies back the use of standardized communication in resuscitation maneuvers to improve the communication and care of the patient during life-support maneuvers [15], and other research studies have associated the use of “action-linked phrases” such as “shock delivered, start compressions” with a decreased start time of the chest compressions [16].

The general objective of our study was to analyze the quality of the CPR, performed by individuals who had no prior knowledge on resuscitation, after receiving a set of instructions (structured and unstructured/intuitive) from an expert within a context of simulated CPR. The specific objective was to design a simple and structured method for the fast learning of CPR on-site.

## 2. Materials and Methods

### 2.1. Study Design and Settings

A post-intervention two-group post-test-only randomized experiment [17,18,19,20] was designed, in which the experimental group (EG) received standardized instructions, and the control group (CG) received intuitive, unstructured instructions. This is one of the simplest experimental designs [21]. The groups (experimental and control) were randomly assigned. One group received the training; the other group did not receive the training, and was used for comparison. A previous trial was not required for this design, as participants were randomly assigned to the groups, and thus it was assumed that both groups were statistically equivalent. In this design, the objective was to determine whether differences existed between the two groups after the training program. Therefore, a prior trial was not considered for this study design. Although this test could have been used to determine whether the groups were comparable before the experiment, this was not done, to avoid the possible negative effects entailed by the trail to the internal validity, which could be detrimental for the participants’ learning. On the other hand, the participants were randomly assigned to the groups and the experimental conditions, ensuring that the groups were equivalent (they had the same socio-demographic characteristics, particularly not having any previous training or prior knowledge about resuscitation).

### 2.2. Selection of Participants

The target population was the university population from the Region of Murcia (Spain); volunteers were solicited through announcements in the virtual campus among the students from the Catholic University of Murcia. The collection of data was performed between the months of November 2019 and February 2020.

The study included all the volunteer participants older than 18 who had signed the informed consent form and who did not comply with the exclusion criteria. These exclusion criteria were: physical limitation that could impede them from performing chest compressions and ventilation for 2 min, intellectual limitation that could impede them from following and performing the orders, refusing to participate in the study, being a healthcare worker or a healthcare student, and having received CPR training at least 5 years prior. The participants were informed about the purpose of the study to evaluate the efficacy of a method to teach CPR on-site in the least amount of time. The participants were not informed about the results of their intervention until the end of the study. The expert resuscitators were selected from clinical simulation teaching staff volunteers at the university. The inclusion criteria were: being an instructor in basic life support (BLS) and automated external defibrillation (AED) accredited by the European Resuscitation Council (ERC), and/or being a CPR professor for more than 2 years. Ultimately, this group was comprised of eight experts, of which six were women and two were men.

### 2.3. Intervention

After complying with the eligibility criteria to participate in the study, the participants were randomly assigned to the experimental group “structured orders” or to the control group “unstructured orders”. This assignment was performed using a random assignment tool (sealedenvelope.com, London, UK). Likewise, once assigned to a group, the roles of ventilation (VR) or chest compression (CR) were randomly assigned. Participants were blinded to the allocation until randomization. For performing the mouth-to-mouth ventilations, face protection devices (Laerdal^®^ Face Shield, Laerdal Medical Corporation, Stavanger, Norway) were made available to the participants. When the research was conducted, knowledge about the COVID-19 pandemic was unknown in Spain.

**Figure 1:**
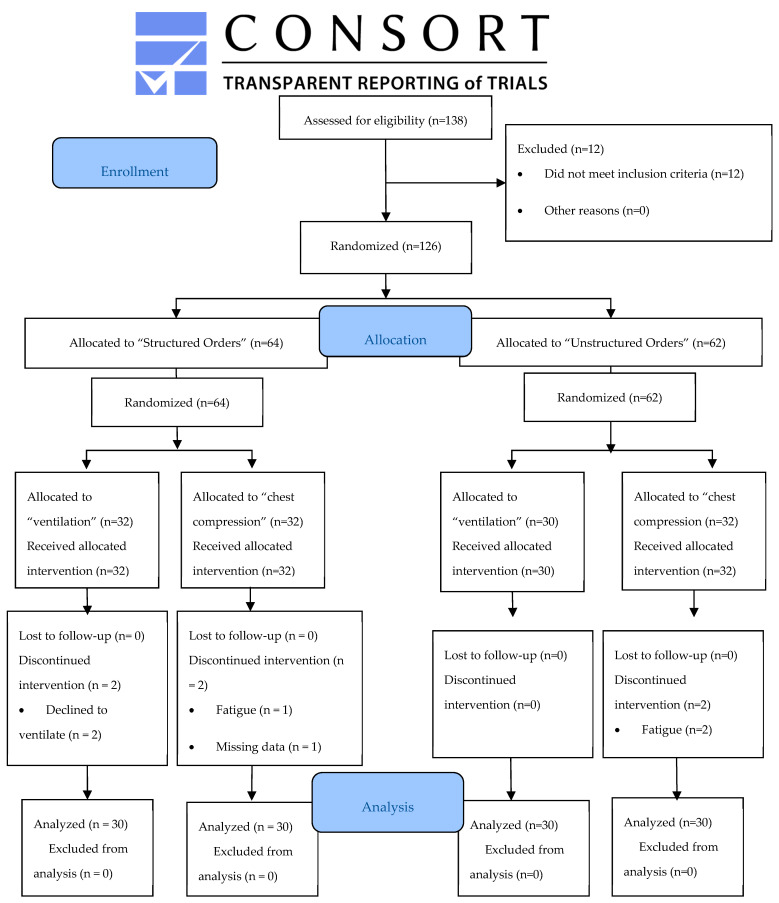
Study CONSORT flow diagram.

A total of 138 individuals comprised the final sample. Of these, 12 were excluded because they had received CPR training in the last 5 years. Of the remaining 126 participants, 64 were randomly assigned to the structured orders group (experimental group, EG) and 62 to the unstructured orders group (control group, CG). Of the 64 EG participants, 32 were assigned the ventilation role (VR) and 32 to the chest compression role (CR); after the randomization, two VR participants were excluded when they refused to perform mouth-to-mouth ventilation, one CR participant was excluded for fatigue which impeded concluding the trial, and one participant was excluded due to loss of data. From the 62 participants in the GC group, 30 were assigned to the VR group and 32 to the CR group; after the randomization, two CR participants were excluded due to fatigue that impeded them from finishing the trial. Lastly, the 120 remaining participants received the training planned, and the results were analyzed. A CONSORT study flow diagram is shown in Figure 1.

### 2.4. Experimental Group: Structured Orders

Before starting, the participants were told that they were dealing with a simulated scenario on the street, and that they were witnessing how an expert performed CPR on a person in OHCA. They were told that the expert would ask for their help after two cycles of 30 compressions/2 ventilations (30:2) after their arrival. After this period, they would receive a series of orders that they had to follow to provide CPR for the next 2 min.

Only compressions were performed during the time the expert gave orders. The orders provided (adapted from the main guides in CPR terminology) [16,22,23,24,25] to the participants with the chest compressions role (CR) were:

1. “Kneel in front of me”.
2. “Interlock your hands, one over the other, and straighten your arms”.
3. “When I count to three, you have to place your hand in the middle of the chest and compress “hard and fast” [25] 30 times”.
4. “Have you understood?” (If the participant answered “NO” to this question, the order would be repeated again without any changes).
5. “1, 2, 3, now!”

The orders provided to the participants with the ventilation role (VR) were given, while the CR participants performed the first chest compressions. These orders were:

1. “Put your hand on the forehead”.
2. “Put the other hand on the chin and lift”.
3. “Cover the nose and blow two times when your partner reaches 30”.

In this sequence of instructions, to warn the rest of the participants and the resuscitators that the end of the compressions was near and to minimize the interruptions, the expert had the added requisite of counting the last five compressions aloud.

The expert who participated in the experimental group was trained in the structured learning method, and had previously practiced in simulation. The expert was the same for the completely experimental group.

### 2.5. Control Group: Structured Orders

To conduct the trial with the unstructured orders group (CG), the expert and the participants were informed about the same simulation scenario. The experts were told that they could ask for help after two cycles of 30:2, and they were asked to give the sequence of orders they thought to be faster and more opportune, following their intuition and previous knowledge, in order for the participants to relieve them in the following 2 min.

To eliminate the learning effect [13], meaning the familiarization of the experts with the procedure, and therefore improving the results in later trials, the pretest trials were dispensed with [26], and eight different experts were selected to interact with the CG participants, so that they were not allowed to repeat the trial more than four times. The experts were not study participants, and they did not have prior knowledge of the structured orders utilized with the EG or the hypothesis of the study.

Comments, corrections, or explanations were not allowed in any of the two groups once the orders were given and the CPR started by the participants.

### 2.6. Analysis Parameters

The main result of the study was the time needed for giving orders (seconds). The secondary result variables were: pauses between compression and ventilation (seconds), depth (mm), rate (compressions/minute), effective rate of ventilation (% between 500–600 mL), rate of chest recoil (%), positioning of the hands (% of success with respect to the center of the chest), scores reached in the mannequin (compression score (0–100), of ventilation (0–100), total/final (0–100)), age (years), sex (male/female), weight (kg), height (meters), and body mass index (BMI) (kg/m2).

### 2.7. Measurements

The demographic data were collected in questionnaires after the informed consent. For collecting the data relative to the quality of the CPR, the Resusci Anne QCPR^®^ manikin (Laerdal Medical Corporation, Stavanger, Norway) was utilized, which was calibrated and checked before conducting the experiment, and periodically during the experimental phase.

### 2.8. Analysis

The continuous variables with a normal distribution were expressed as mean and standard deviation (SD); the data without a normal distribution were described as median and interquartile range (IQR). A difference in means analysis was performed for independent samples with the Student’s *t* test or the Mann–Whitney U test, according to the distribution of the data. The categorical variables were described as frequencies and percentages (%). The results were considered statistically significant at *p* < 0.05. The data were reported according to the CONSORT guidelines [27]. The processing and analysis of the data were conducted with the statistical package IBM SPSS^®^ for Windows version 22.0 (IBM Corporation, Armonk, DA, USA).

### 2.9. Ethical Considerations

All the participants gave their informed consent for inclusion before participating in the study. The study was conducted in accordance with the Declaration of Helsinki, and the protocol was approved by the Ethics Committee of Catholic University of Murcia (CE031901).

## 3. Results

The mean age of the participants was 21 years old (SD 4.75), the mean height was 1.71 m (SD 0.08), the mean weight was 66.1 kg (SD 12.14), and the mean BMI was 22.5 kg/m^2^ (SD 2.92). Of these, 60% (n = 72/120) of the participants were women. Table 1 shows the demographic data according to groups (CG and EG).

**Figure 2:**
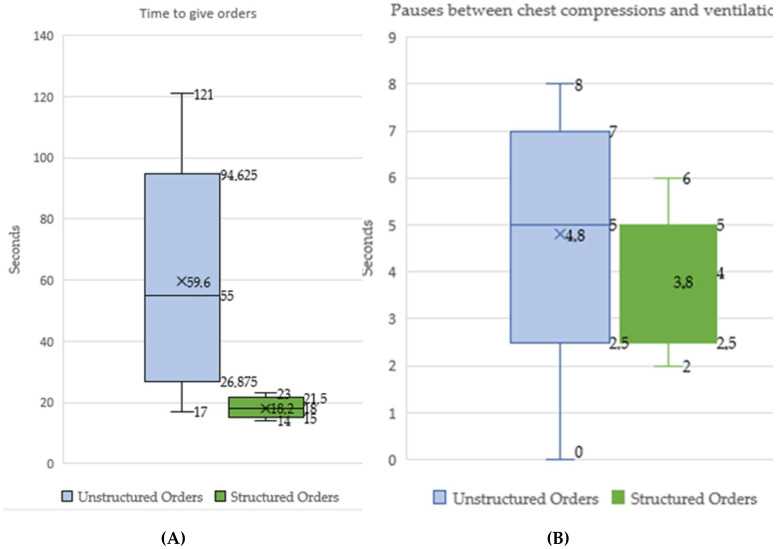
(**A**) Time needed to give orders. (**B**) Pauses between chest compressions and ventilations.

Statistically significant differences were obtained (*p* < 0.0001) between the EG and CG for the variables: time needed to give orders (Figure 2A), pauses between chest compressions and ventilations (Figure 2B), depth, overall score, chest compression score, and chest recoil.

The mean depth was 51.1 mm (SD 7.94) for EG, and 42.2 mm (SD 12.04) for the CG (Table 2).

The median of the variable rate for the EG was 121 compressions per minute (IQR 110, 130), and 121 compressions per minute (IQR 113, 132) for the CG. The median of effective ventilation (% between 500–600 mL) was 20% (IQR 5, 50) for the EG, and 40% (IQR 0, 60.42) for the CG. The median for chest recoil was 86.32% (IQR 62.36, 98.87) for the EG, and 58.3% (IQR 27.46, 84.33) for the CG. From the participants, 83.3% (25/30) correctly positioned their hands in the EG, and 80% (24/30) in the CG (Table 2).

No statistically significant differences were found according to sex, age, height, weight, or BMI between the CG and the EG, or the VR or CR groups.

**Figure 3:**
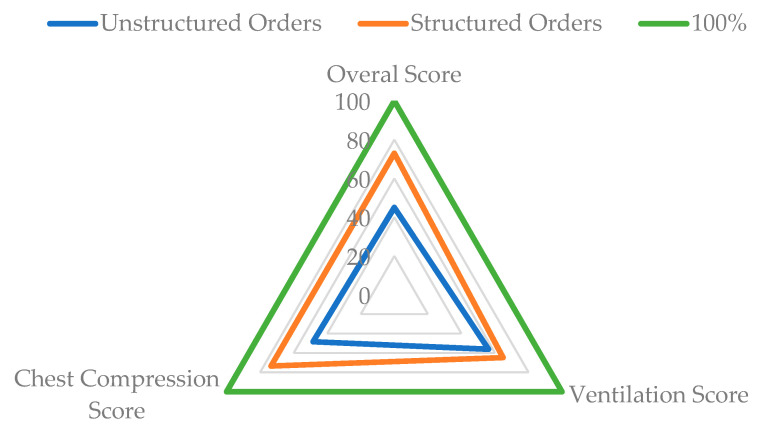
Comparison of the scores reached by each group, with respect to the maximum score.

As for the scores achieved (Figure 3), the means/medians obtained were: 67 points (IQR 49, 83) for the EG, and 65 points (IQR 34, 81.5) for the CG in ventilation; 78.5 points (IQR 61.5, 87.25) for the EG, and 53 points (IQR 37.25, 61) for the CG in compression; 73 points (SD 14.5) for the EG, and 45 points (SD 22.47) for the CG in the overall CPR score.

As for ventilation, 40% (12/30) of the CG participants correctly performed the head-tilt/chin-lift maneuver to ventilate. In the EG, this maneuver was performed by 66.67% (20/30) of the participants. Statistically significant differences were found in effective ventilation after performing the head-tilt/chin-lift maneuver (*p* < 0.0001).

## 4. Discussion

Increasing the rates of resuscitation when witnessing cardiorespiratory arrests is still a challenge in Spain [28]. This study intended to demonstrate the efficiency of a fast and structured method of communication on-site for situations in which an expert is performing CPR in the presence of bystanders who are willing to provide relief, either in the compression, or in ventilation maneuvers.

Observation-based, vicarious, or through-demonstration learning [13] are some one of the most-commonly utilized methods for learning motor skills [29]. Diverse studies have suggested that visual orientation can accelerate the acquisition of complex motor skills [30]. In our case, we believe that this visual guide offered advantages over instructions that were not provided in person (such as through the phone, for example). We also believe that observational learning had effects (in both the experimental groups as well as the control group) on the quality of resuscitation; this is the reason why the co-variation of the results attributed to the independent variable (structure method) is even more powerful, as this observational learning was found in both groups (experimental and control). Therefore, it is thought that there could be a causal attribution of the structured method towards the improvement of CPR. Thus, it can be concluded that the use of structured orders results in the better performance of CPR, compared to intuitive or unstructured orders of individuals without prior knowledge of CPR.

The main finding of our study consisted of the statistically significant decrease in the time needed to provide the orders that allowed relieving the expert and performing high-quality CPR. The time invested by the expert in the experimental group was significantly less compared to the unstructured orders group. The pauses between compressions and ventilations also decreased. In addition, an improvement was registered in the mean depth and the chest recoil, compared to the set of unstructured orders.

From our point of view, the decrease in these times could be related to the simplicity of the orders, in agreement with the results from Hunt et al. [16], insofar as it was suggested that there was a greater chance that the appropriate action could occur when short, easy, and specific phrases were utilized. The pauses between compression and ventilation were significantly reduced in the experimental group, with this result possibly due to the implementation of improvements proposed by Lauridsen et al. [15], such as the use of backwards counting before the relief, or counting out loud of the last five compressions.

The simplification of the orders can also improve the quality of the chest compressions [31]. This could help in the understanding and discussion of the results from other studies [32] that suggest that non-trained bystanders are not useful during CPR.

As for ventilation, achieving the volunteer’s relief of the compression expert relies on a margin of 30 compressions (between 15–17 s) for the ventilation volunteer to receive the indications correctly. The participants who performed the head-tilt/chin-lift maneuver performed effective ventilations (500–600 mL) that were significantly better than those who did not. However, for the ventilation skills, no significant differences were found between the groups; in fact, the scores reached by the unstructured orders group were higher. Thus, it seems possible that although a layperson resuscitator performs the head-tilt/chin-lift maneuver correctly, this does not ensure proper ventilation, but could result in mistakes in the volume ventilated (hyperventilation or hypoventilation).

This makes us believe that on the one hand, ventilation is a skill that requires more training time compared to compression, and on the other hand, that compression-only CPR in the presence of an expert is also a valid alternative; not only for those people who are reticent about performing mouth-to-mouth ventilation [33], but also for the notable public interest in learning CPR [34]. All of these improvements resulted in higher scores, provided by the simulator, of the experimental group than the control group.

The variables rate and hand positioning did not show significant differences between both groups, and were within the range accepted by scientific societies [35].

Our study was conducted with the aim of assessing the effectiveness of providing orders. However, this method has an added benefit, in that in a real-life situation in which the expert has to be relieved, a layperson could be properly corrected fast. Thus, during the resuscitation procedure, the strong points could be emphasized, and the weak points improved, in order to provide high quality CPR until the arrival of the emergency services. This advantage is shown in studies, such as those from González-Salvado et al. [36], which affirms that practical learning guided by an instructor provides better results.

In conclusion, the learning of technical skills seems to be greatly simplified when the layperson or beginner is allowed to observe an expert, as pointed out in other studies [37,38], which associate a greater ability of response and performance of the layperson after viewing ultra-brief videos.

### Limitations

Among the main limitations of the study, we find the extrapolation of the results due to the characteristics of the sample and the type of experiment. The external validity could be improved with a representative sample of a real population, not only with a healthy university population. In the second place, we believe that the validity of the experiments with simulation is limited, as it does not occur in a real-life context, and other variables, such as stress or the interference from other spectators, could not be taken into account. Another limitation that should be mentioned is the lack of a pre-test to assess the resuscitation skills of the participants, beyond their statement of being a layperson. In this case, the design of the study would have been more complex. Thus, this was not done, to avoid the threat to the internal validity known as learning. However, if properly managed, it could have provided a greater internal validity of the study, which could have helped establish, with greater precision, if the groups were homogeneous for their comparison.

## 5. Conclusions

The use of a sequence of simple, short and specific orders, together with observation-based learning, makes possible the execution of chest compression maneuvers that are very similar to those performed by rescuers, and allows the teaching of the basic notions of ventilation.

Improvements were identified in the variables “time needed to give orders”, “pauses between chest compressions and ventilations”, and “chest recoil” when CPR was performed by laypersons in the experimental group.

The method of structured orders was shown to provide an on-site learning opportunity when faced with the need to maintain high-quality CPR in the presence of an expert resuscitator before the arrival of emergency services, and after their arrival, in case further help is needed.

More research studies are needed to assess these findings in a non-university population, and to measure other variables that could have an influence on the results, such as the role of the layperson’s stress when facing this situation in a non-simulation context.

## Figures and Tables

**Table 1:** Baseline demographics (n = 120).

Demographic Variables	Unstructured Orders Group (n = 60)	Structured Orders Group (n = 60)	*p*-Value
Age (years)—mean (SD)	21 (5.07)	21 (3.78)	0.669
Sex (% female)	36 (60)	36 (60)	0.574
Height (m)—mean (SD)	1.72 (0.09)	1.70 (0.09)	0.31
Weight (kg)—mean (SD)	67.3 (13.46)	64.3 (11.68)	0.209
BMI (kg/m^2^)—mean (SD)	22.7 (3.20)	22.1 (2.77)	0.236

**Table 2:** Study outcome data.

Variables of Study	Unstructured Orders	Structured Orders	*t*-Test/Mann–Whitney U	*p*-Value
Time needed to give orders (seconds)—mean (SD)	55.3 (26.09)	17.9 (2.34)	*t* = 7.813	<0.001
Pauses between chest compressions and ventilations (seconds)—median (IQR)	5 (5, 6)	4 (3, 4)	U = 152	<0.001
Depth (mm)—mean (SD)	42.2 (12.04)	51.1 (7.94)	*t* = 3.38	0.001
Rate (comp/min)—median (IQR)	121 (113, 132)	121 (110, 130)	U = 443.5	0.923
Overall score (0–100)—mean (SD)	45.03 (22.5)	73 (14.5)	*t* = 5.73	<0.001
Ventilation score (0−100) ^a^—median (IQR)	65 (34, 81.5)	67 (49, 83)	U = 370.5	0.437
Chest compression score (0–100)—median (IQR)	53 (37.25, 61)	78.5 (61.5, 87.25)	U = 171	<0.001
Hands position—median (IQR)	100 (100, 100)	100 (100, 100)	U = 437.5	0.784
% Effective ventilation (500–600 mL) ^a^—median (IQR)	40 (0, 60.42)	20 (5, 50)	U = 394.5	0.682
% Chest recoil—median (IQR)	58.3 (27.46, 84.33)	86.32 (62.36, 98.87)	U = 263.5	0.006

^a^ Two cases with missing data.
